# RNA SARS-CoV-2 Persistence in the Lung of Severe COVID-19 Patients: A Case Series of Autopsies

**DOI:** 10.3389/fmicb.2022.824967

**Published:** 2022-01-31

**Authors:** Tamara Caniego-Casas, Laura Martínez-García, Marina Alonso-Riaño, David Pizarro, Irene Carretero-Barrio, Nilda Martínez-de-Castro, Ignacio Ruz-Caracuel, Raúl de Pablo, Ana Saiz, Rosa Nieto Royo, Ana Santiago, Marta Rosas, José L. Rodríguez-Peralto, Belén Pérez-Mies, Juan C. Galán, José Palacios

**Affiliations:** ^1^Pathology Department, Hospital Universitario Ramón y Cajal, Madrid, Spain; ^2^Instituto Ramón y Cajal for Health Research (IRYCIS), Madrid, Spain; ^3^CIBERONC, Instituto de Salud Carlos III, Madrid, Spain; ^4^Microbiology Department, Hospital Universitario Ramón y Cajal, Madrid, Spain; ^5^Centro de Investigación Biomédica en Red en Epidemiología y Salud Pública, Madrid, Spain; ^6^Pathology Department, Hospital Universitario 12 de Octubre, Madrid, Spain; ^7^Instituto 12 de Octubre for Health Research, Madrid, Spain; ^8^Faculty of Medicine, Complutense University, Madrid, Spain; ^9^Faculty of Medicine, University of Alcalá, Alcalá de Henares, Spain; ^10^Anaesthesiology and Surgical Critical Care Department, Hospital Universitario Ramón y Cajal, Madrid, Spain; ^11^Medical Intensive Care Unit, Hospital Universitario Ramón y Cajal, Madrid, Spain; ^12^Respiratory Department, Hospital Universitario Ramón y Cajal, Madrid, Spain

**Keywords:** COVID-19, viral persistance, autopsy, RNA, immunosuprression

## Abstract

The exact role of viral replication in patients with severe COVID-19 has not been extensively studied, and it has only been possible to demonstrate the presence of replicative virus for more than 3 months in a few cases using different techniques. Our objective was to study the presence of RNA SARS-CoV-2 in autopsy samples of patients who died from COVID-19 long after the onset of symptoms. Secondary superimposed pulmonary infections present in these patients were also studied. We present an autopsy series of 27 COVID-19 patients with long disease duration, where pulmonary and extrapulmonary samples were obtained. In addition to histopathological analysis, viral genomic RNA (gRNA) and viral subgenomic RNA (sgRNA) were detected using RT-PCR and *in situ* hybridization, and viral protein was detected using immunohistochemistry. This series includes 26 adults with a median duration of 39 days from onset of symptoms to death (ranging 9–108 days), 92% of them subjected to immunomodulatory therapy, and an infant patient. We detected gRNA in the lung of all but one patient, including those with longer disease duration. SgRNA was detected in 11 out of 17 patients (64.7%) with illness duration up to 6 weeks and in 3 out of 9 patients (33.3%) with more than 6 weeks of disease progression. Viral protein was detected using immunohistochemistry and viral mRNA was detected using *in situ* hybridization in 3 out of 4 adult patients with illness duration of <2 weeks, but in none of the 23 adult patients with an illness duration of >2 weeks. A remarkable result was the detection of viral protein, gRNA and sgRNA in the lung cells of the pediatric patient after 95 days of illness. Additional pulmonary infections included: 9 acute bronchopneumonia, 2 aspergillosis, 2 cytomegalovirus, and 1 BK virus infection. These results suggest that in severe COVID-19, SARS-CoV-2 could persist for longer periods than expected, especially in immunocompromised populations, contributing to the persistence of chronic lung lesions. Additional infections contribute to the fatal course of the disease.

## Introduction

COVID-19 is described as a disease with a biphasic course and three stages (early infection, pulmonary, and hyperinflammatory stage) (Siddiqi and Mehra, 2020). The initial phase is characterized by intense viral replication and high viral load, followed by a strong and dysregulated inflammatory response. Nonetheless, it is difficult to specify the duration of each phase. Over the first week, in those patients with mild or moderate COVID-19 disease, the viral load decreases; Conversely, in critical patients, high viral loads have been quantified up to 2 weeks using real-time RT-PCR, and detected by immunohistochemistry (IHC) and *in situ hybridization* (ISH; Schaefer et al., 2020). Several studies showed that viral RNA could be detected using RT-PCR for periods longer than 2 months (Avanzato et al., 2020; Reuken et al., 2021), but the presence of viral RNA and protein was difficult to demonstrate using ISH and IHC, respectively, after 2 weeks (Evert et al., 2021). Most autopsy studies have been performed in patients with <30 days of illness; and therefore, the exact duration of viral RNA presence in lung tissue has not been described comprehensively.

In addition to SARS-CoV-2 infection, patients with severe COVID-19, especially the immunocompromised and those with longer ICU stays are prone to develop other pulmonary infections (Evert et al., 2021), including acute bronchopneumonia, reactivation of herpesviruses such as cytomegalovirus (CMV) and fungal infections, whose frequencies vary among different autopsy series.

Here, we study the presence of RNA SARS-CoV-2 after long periods of illness, using a collection of post-mortem specimens from lungs and extra-respiratory organs. We detected genomic SARS-CoV-2 RNA (gRNA) and subgenomic RNA (sgRNA) in 27 patients with a median time to death of 39 days. In addition, we evaluated the presence of other pulmonary infections in these patients with severe COVID-19.

## Materials and Methods

### Autopsies

The Research Ethics Committee approved the study (reference: Necropsias_Covid19; 355_20). It included all consecutive autopsies of COVID-19 patients performed at Hospital Universitario Ramón y Cajal (Madrid, Spain) from April 2020 to March 2021 (*n* = 26). The autopsies corresponded to patients with severe respiratory disease and were requested by the medical staff according to clinical interest and represented about 3% of all COVID-19 deaths during this period. Consequently, the series does not represent the complete spectrum of causes of death attributable to COVID-19. All autopsies were performed under the consent of the patients’ relatives and carried out following safety protocols, in a negative pressure autopsy room, using personal protection equipment, as previously reported (The COVID-19 Autopsy Project, 2020). All autopsies were performed in less than 24 h after the patient’s death and included both gross and histologic examination of the organs.

In the first 14 consecutive decedents, because of biosecurity concerns, we took in-corpore representative sections from the heart, lungs, liver, kidney, pancreas, and bone marrow. Due to improved technical training regarding handling of instruments and sampling collection while wearing personal protection equipment, in the rest of the patients we extracted the complete heart and lung block, left kidney, spleen, and sections from the liver, pancreas, and bone marrow. One autopsy was limited to the lungs, as requested under the consent of the patient’s relatives.

### Histopathological Evaluation

After fixation in 10% buffered formalin for 24–48 h, samples from the five pulmonary lobes were taken in all patients. All histological evaluations were blinded to clinical data. The histopathological classification of the diffuse alveolar damage (DAD) lesions was performed according to Li et al. (2021), as previously reported (Pérez-Mies et al., 2021).

In addition to hematoxylin and eosin stain, PAS and Grocott stains were performed in suspicious cases to highlight fungal infections. Immunohistochemistry for Herpesvirus (clone 10A3, Roche), CMV (clone CCH2 + DDG2, Agilent) and polyomavirus BK (anti-SV40, clone Pab416, Gennova) were performed on a Dako Omnis platform and the staining was visualized with the EnVision system FLEX/HRP (Agilent, Santa Clara, CA, United States). These cases were selected by morphological features in lung samples suspicious of those infections.

### SARS-CoV-2 RNA Detection by RT-PCR

SARS-CoV-2 detection was done in post-mortem swabs taken from the nasopharynx (NPS), lungs (LS) (the two superior lobes), heart, liver, and right kidney. In addition, testing was done in post-mortem formalin-fixed paraffin-embedded (FFPE) tissue from the lungs (all lobes), heart, liver and kidney in all patients, and large intestine in eight patients. RT-PCR was utilized for detection of genomic SARS-CoV-2 RNA (gRNA) and subgenomic viral RNA (sgRNA) in all patients.

For gRNA detection, swab samples were sent on the same day to the Microbiology Department for the detection of genomic SARS-CoV-2 RNA. RNA extraction and RT-PCR amplification were performed within 3 h after reception in the laboratory. RNA extraction was performed using MagmaxTM Core Nucleic Acid Purification Kit (Thermo Fisher, Waltham, MA, United States) and gRNA SARS-CoV-2 was detected using TaqmanTM 2019 nCoV assay (Thermo Fisher, Waltham, MA, United States). Samples with a cycle threshold (Ct) lower than 40 were considered positive.

For FFPE samples, RNA was extracted from 10 sections of 5 μm obtained from paraffin blocks using RecoverAll Total Nucleic Acid Isolation Kit (Invitrogen), following the manufacturer’s instructions. RNA quantity was measured fluorometrically with Qubit RNA high-sensitivity assay kit (Invitrogen, Waltham, MA, United States). gRNA SARS-CoV-2 was detected in the same way as the swab samples, using TaqmanTM 2019 nCoV assay (Thermo Fisher, Waltham, MA, United States). Samples with a Ct lower than 40 were considered positive.

sgRNA was detected using RT-PCR. Retrotranscription was performed with High-Capacity cDNA Reverse Transcription Kits (Thermo Fisher Scientific), following the manufacturer’s instructions. Specific primers were designed between the leader region and the nucleocapsid protein (N) gene (forward primer sequence: 5′ ACCTTCCCAGGTAACAAACCA 3′; reverse primer sequence: 5′ GGTCCACCAAACGTAATGCG 3′; amplicon size 129 bp). The PCR was performed at 54° annealing temperature for 45 cycles. PCR products were run with High Sensitivity D1000 ScreenTape kit on the Tape Station 2200 (Agilent, Santa Clara, CA, United States). Confirmation of positive fragments was performed using Sanger sequencing.

As internal quality controls, RNAseP or MS2 were used. Only those PCR assays yielding a positive amplification for internal controls were validated following the manufacture’s guidelines.

### SARS-CoV-2 Immunohistochemistry and *in situ* Hybridization

In each patient, the FFPE RT-PCR positive lung sample with the lowest Ct value was also analyzed with IHC and ISH.

For SARS-CoV-2 immunohistochemistry, we used the monoclonal antibody (1A9, Genetex Inc., Irvine CA, United States) against the spike protein of SARS-Cov/SARS-CoV-2 in 1:100 dilution on 3 μm slides obtained from paraffin blocks. Antigen retrieval was performed with 10 mM citrate buffer (pH 6.0). The staining was visualized with the EnVision system FLEX/HRP (Agilent, Santa Clara, CA, United States).

RNA ISH for SARS-CoV-2 was performed using the RNAscope^®^ SARS-CoV-2 probes for the SARS-CoV-2 S gene encoding the spike protein (catalog #848561, Advanced Cell Diagnostics, Inc., Hayward, CA, United States) on a Leica Bond III automated stainer (Leica Biosystems, Wetzlar, Germanyı), according to the manufacturer’s instructions. Briefly, 4-μm formalin-fixed and paraffin-embedded tissue sections were pre-treated with heat and protease prior to hybridization. Tissue sections were hybridized separately with the target probe to detect infected cells and with the positive and negative control probes. Specific staining signals were identified as brown, punctate dots present in the cytoplasm.

### Ultrastructural Study

We performed a transmission electron microscopic examination of the FFPE tissue from the lung of the pediatric patient. Thin sections were stained with uranyl acetate and lead citrate and were examined with an EM-10 Zeiss microscope.

## Results

### Demographic and Pathological Findings

This series included 26 adults and 1 pediatric patient. The clinical data of the adult patients are shown in Table 1, including 21 males and 5 females, with a median age of 69.5 years (IQR 14.8) and a median illness duration of 39 days (IQR 13.3).

**TABLE 1:** Clinical characteristics of adult patients.

Demographics and clinical characteristics			Total number of observations
Age, years	Median (IQR)	69.50 (14.75)	26
	Min, max	52, 91	
Gender, *n* (%)	Male	21 (80.77)	26
Weight, kg	Median (IQR)	80 (20)	25
	Min, max	53, 109	
DM, *n* (%)		2 (7.69)	26
Hypertension, *n* (%)		11 (42.30)	26
Patients admitted to ICU, *n* (%)		22 (84.61)	26
Total days	Median (IQR)	39 (13.25)	26
	Min, max	9, 108	
Hospitalization days	Median (IQR)	30.5 (11.75)	26
	Min, max	3, 69	
ICU days	Median (IQR)	25.50 (16)	22
	Min, max	12, 95	
Mechanical ventilation, *n* (%)		22 (84.62)	26
Corticosteroids use, *n* (%)		24 (92.31)	26
Immunomodulatory therapy*, *n* (%)		23 (88.46)	26

*Including tocilizumab and/or interferon β1a.

**FIGURE 1:**
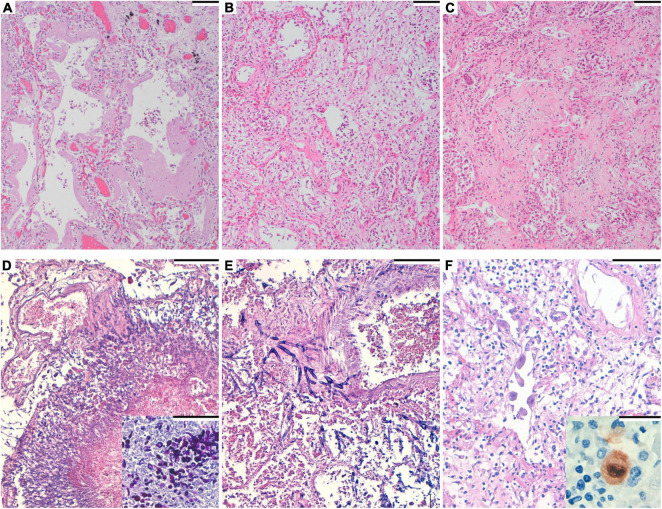
**(A)** Hematoxylin and eosin. Diffuse alveolar damage (DAD), exudative phase with hyaline membranes formation. Scale bar: 100 μm. **(B)** Hematoxylin and eosin. DAD, organizing phase. Scale bar: 100 μm. **(C)** Hematoxylin and eosin. DAD, fibrotic phase. Scale bar: 100 μm. **(D)** Hematoxylin and eosin. Numerous filamentous fungi are seen in the lung. Scale bar: 100 μm. Inset: Periodic acid–Schiff staining, highlighting the fungi. **(E)** Hematoxylin and eosin. Filamentous fungi invading the arteriolar wall in angioinvasive aspergillosis. Septate hyphae with dichotomous branching at acute angles of around 45° are apparent. Scale bar: 100 μm. **(F)** Hematoxylin and eosin. Endothelial pulmonary cells with viral cytopathic changes. Scale bar: 100 μm. Inset: Immunohistochemistry for cytomegalovirus, showing positive staining.

Histopathological analysis revealed severe diffuse alveolar damage (DAD) in all but one patient, in whom only occasional areas of hyaline membranes were found. The predominant lung pattern was DAD in fibroproliferative stage, but exudative or fibrotic lesions were also present in different proportions in each patient (Figures 1A–C and Table 2).

**TABLE 2:** Lung pathological findings.

			Total number of observations
Patients with predominant pattern, *n* (%)	Normal lung	1 (3.85)	26
	Exudative DAD	6 (23.07)	26
	Proliferative/organizing DAD	15 (57.69)	26
	Fibrotic DAD	4 (15.38)	26
Vascular thrombi, *n* (%)		18 (69.23)	26
Endothelialitis, *n* (%)		11 (42.31)	26
Infections, *n* (%)			
	Acute bronchopneumonia	11 (42.31)	26
	Aspergillosis	2 (7.69)	26
	Cytomegalovirus	2 (7.69)	26
	Subpleural abscess	1 (3.85)	26

Under histological examination, lesions of acute bronchopneumonia were observed in 11 patients (42.3%). Moreover, we observed the presence of hyphae within areas of mixed inflammatory infiltrates in two patients. These hyphae were visible under the PAS and Grocott techniques (Figures 1D,E) and were also present in the parenchyma of the heart and kidney of one patient. In one case fungi were identified as *Aspergillus flavus* using PCR (partial sequencing of 18S rDNA). The fungal structures of the other case were not identified by PCR nor culture. We observed intranuclear and intracytoplasmic inclusions in respiratory epithelial cells, suggestive of CMV infections, in 2 patients (7.7%). Specific immunohistochemistry for CMV was performed and rendered positive results (Figure 1F). We did no detect CMV inclusions in any other organ. The clinical characteristics of patients with CMV and aspergillosis diagnosis are specified in Table 3.

**TABLE 3:** Features of patients with a cytomegalovirus (CMV) or aspergillosis diagnosis.

	Patient 6	Patient 7	Patient 8	Patient 12
Age (years)	60	68	72	55
Pathological CMV and/or aspergillosis diagnosis	CMV	Aspergillosis	CMV	Aspergillosis
Clinical CMV and/or aspergillosis diagnosis	No	*Aspergillus flavus*	CMV	No
Illness duration (days)	32	32	34	37
Hospitalization duration (days)	29	18	26	30
UCI stay (days)	19	12	25	30
Comorbidities	None	Oropharyngeal squamous cell carcinoma, former smoker, stroke	Bladder carcinoma, chronic heart disease	None
Corticosteroids use	Yes	Yes	Yes	Yes
Immunomodulatory therapy (Tozilizumab)	Yes	Yes	Yes	Yes

*ICU, intensive care unit; CMV, cytomegalovirus.*

Other pathological findings in the lungs, hearts, kidneys, livers, and bone marrows are enumerated in Table 4.

**TABLE 4:** Main pathological findings in other organs.

		Total number of observations
**Heart**		
Coronary artery atherosclerosis, *n* (%)	4 (16)	25
Left ventricle hypertrophy, *n* (%)	3 (12)	25
Chronic epicardial inflammation, *n* (%)	1 (4)	25
Myocarditis, *n* (%)	1 (4)	25
Senile amyloidosis, *n* (%)	1 (4)	25
**Liver**		
Centrolobulillar necrosis, *n* (%)	4 (16)	25
Esteatosis, *n* (%)	7 (28)	25
Cirrhosis, *n* (%)	1 (4)	25
**Kidney**		
Ischemic necrosis, *n* (%)	8 (32)	25
Acute tubular necrosis, *n* (%)	8 (32)	25
**Bone marrow**		
Haemophagocitosys, *n* (%)	19 (76)	25
Hyperplasia, *n* (%)	22 (88)	25

**FIGURE 2:**
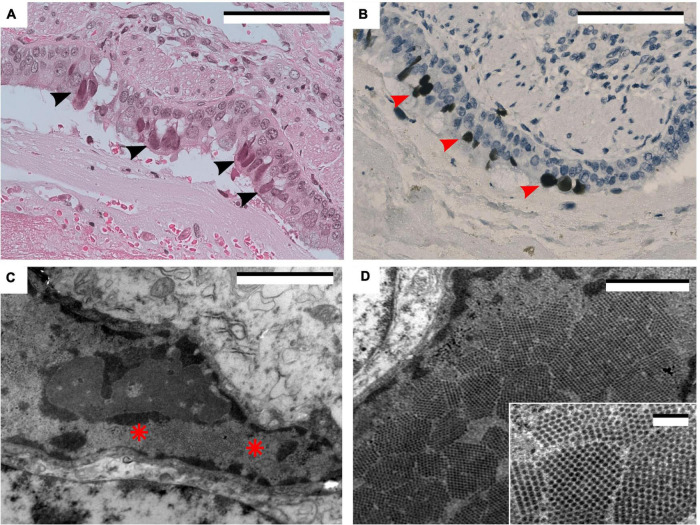
**(A)** Hematoxylin and eosin. Ciliated bronchial epithelial cells with amphophilic nuclear inclusions (arrowheads). Scale bar: 100 μm. **(B)** Polyomavirus BK immunohistochemistry. Same area as panel **(A)**, showing positive staining in the nuclear inclusions (arrowheads). Scale bar: 100 μm. **(C)** Electron micrograph of an infected cell containing intranuclear inclusions (between asterisks). Scale bar: 2 μm. **(D)** Intranuclear icosahedral inclusions. Scale bar: 1 μm. Inset: Note the 40 nm particles characteristic of the polyoma virus group. Scale bar: 200 nm.

This series also included an 8-year-old boy with primary immunodeficiency (PI). This patient was subjected to a haploidentical transplant due to a severe PI characterized by an almost total absence of naïve T cells and T cell receptor excision circles (TRECs). A next generation sequencing study did not identify any pathogenic mutations in the genes related to the main severe combined immunodeficiencies. The patient was waiting for a comparative genomic hybridization study to evaluate the possibility of a 22q11 related syndrome. During his stay in the hospital for the diagnosis of his primary disease, SARS-CoV2 was identified in a NPS during a febrile episode with little respiratory symptoms. The patient recovered from the clinical symptoms but the NPS stayed positive. He developed acute respiratory insufficiency 95 days after the initial diagnosis of COVID-19 and eventually died. In the autopsy, the main pulmonary finding was an extensive bilateral pulmonary hemorrhage. Moreover, some bronchial epithelial cells showed amphophilic nuclear inclusions that were positive for Polyomavirus BK immunohistochemistry. Electron microscopic examination revealed multiple intranuclear viral particles arranged in paracrystalline arrays, approximately 40 nm in diameter, with no envelope, typical of Polyomavirus BK (Figure 2).

### SARS-CoV-2 Detection

**FIGURE 3:**
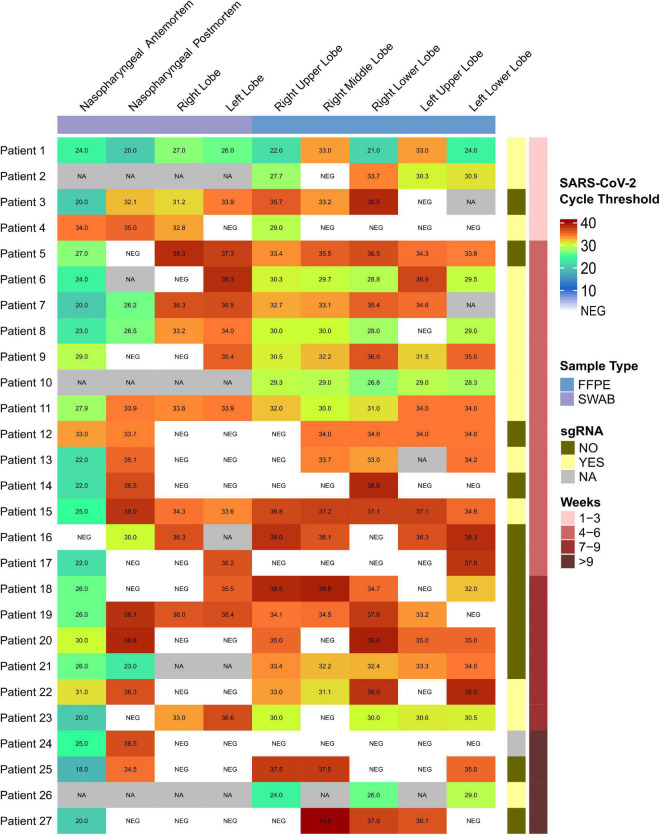
SARS-CoV-2 cycle threshold values heatmap visualization. Upper blue bar indicates sample type (swab or formalin fixed paraffin embedded tissue –FFPE) of nasopharyngeal or lung locations. Right red bar indicates the weeks from onset of symptoms and right yellow bar shows the presence of sub-genomics regions. For antemortem nasopharyngeal swabs, three positive patients did not have the Ct values recorded (NA). One additional patient had a negative result in our hospital, but a positive result in another center. For post-mortem samples, NA indicates no sample available. The infant corresponds to Patient 26.

All patients tested positive for SARS-CoV-2 in NPS during the development of their disease. Ct values are shown in Figure 3. Three patients who tested positive did not have their Ct values recorded, and one additional patient had a negative result in our hospital, but a positive one in another center.

**FIGURE 4:**
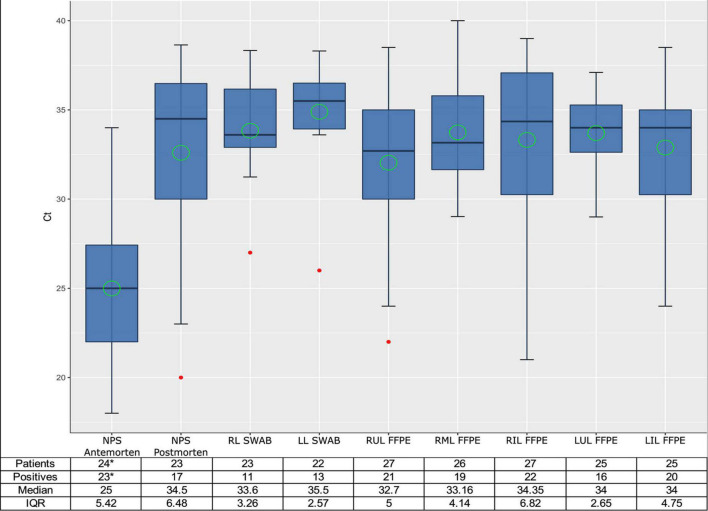
Box plot of SARS-CoV-2 cycle threshold values of the different types of samples. Median is shown as a black line, mean as a green circle and the outliers as red points. ANOVA: Pr(>F) = 4.04e–12***. Ct, cycle threshold; NPS, nasopharyngeal; RL, right lobe; LL, left lobe; RUL, right upper lobe; RML, right medium lobe; RIL, right inferior lobe; LUL, left upper lobe; LIL, left inferior lobe; IQR, interquartile range. *Three positive patients did not have the Ct values recorded. One additional patient had a negative result in our hospital, but a positive result in another Center.

SARS-CoV-2 gRNA was detected in at least one autopsy sample in all patients (Figure 3). The number of samples obtained in the respiratory tract, the number of positive samples for each location, and the median, mean and range Ct values for each location are presented in Figure 4, as well as the comparison of Ct values between premortem swabs and post-mortem samples.

Positive post-mortem NPS were obtained in 17 of 23 (73.9%) patients and positive LS were obtained in 15 of 23 (65.2%) patients. No positive results were obtained in swab samples from the heart, kidney, and liver in all patients except one, who showed a positive result in the heart swab.

**FIGURE 5:**
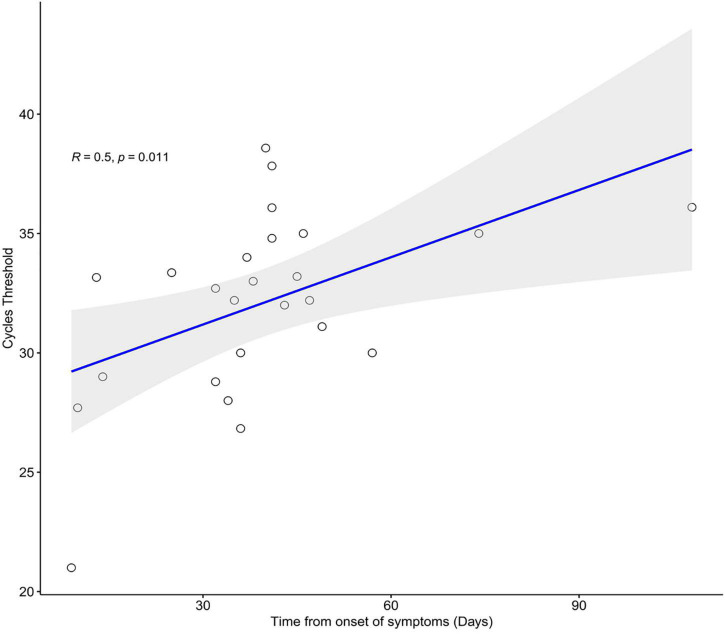
Spearman correlation between days from onset of symptoms and SARS-CoV-2 cycle threshold values of FFPE samples, where regression line is shown in blue and confident interval in gray.

Regarding FFPE samples, positive results were obtained in all but one patient from one or more lung lobes. The frequency of positive SARS-CoV-2 RT-PCR was significantly higher in FFPE lung samples (96.3%) than in post-mortem NPS (73.9%) and LS (65.2%) (*p* = 0.038 and *p* = 0.024, respectively). No statistically significant differences were observed in the Ct values obtained from post-mortem swabs and FFPE positive samples. Ct values from FFPE samples showed a statistically significant positive correlation with illness duration (*r* = 0.5; *p* = 0.011) (Figure 5), indicating lower viral shedding with disease progression.

In adult patients, we detected sgRNA in 11 of 17 (64.7%) patients with illness duration up to 6 weeks. Moreover, sgRNA was also detected in 2 of 9 (22.2%) adult patients with more than 6 weeks of disease progression (Figure 3).

Viral protein – using IHC –, and viral mRNA – using ISH – were detected in 3 out of 4 adult patients with illness duration of less than 14 days, but in none of the 22 patients with an illness duration between 15 and 103 days. Signals were observed in areas with hyaline membranes, in the cytoplasm of pneumocytes, some of them desquamated into the alveolar lumen, and more infrequently in the respiratory epithelium of bronchi or bronchioles. Staining of intraalveolar cells, suggestive of macrophages, was also observed. Staining of endothelial cells was inconclusive in our samples.

**FIGURE 6:**
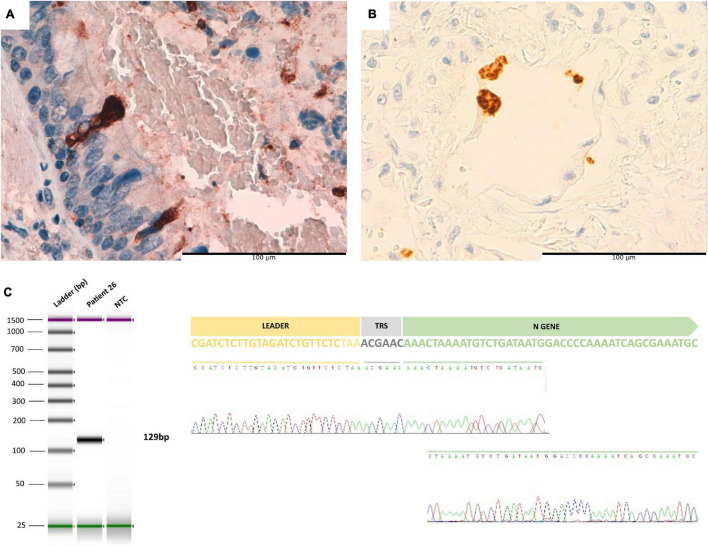
**(A)** Ciliated bronchial epithelial cells positive for SARS-CoV-2 spike protein in patient 26 (immunohistochemistry). **(B)** *In situ* hybridization for SARS-CoV-2 RNA revealed positivity in the cytoplasm of several alveolar cells. **(C)** From left to right: Tape Station report of sgRNA amplification in infant patient. Electropherogram obtained by Sanger sequencing of the amplified product of gRNA where the leader sequence is observed close to the N gene.

We detected viral protein using IHC, viral gRNA using ISH and RT-PCR, and sgRNA in the lung of the pediatric patient after 95 days of the onset of symptoms (Figure 6). We did not observe viral particles suggestive of SARS-CoV2 using electron microscope examination.

## Discussion

To the best of our knowledge, we report the autopsy series with the longest illness duration in which viral detection has been performed in post-mortem lung samples. The availability of post-mortem specimens has provided a comprehensive insight into the pathophysiology of COVID-19. Several autopsy series including 4 to 64 patients (median 16 patients) with a median duration of illness ranging from 5.7 to 38.5 days (median 14.9 days) have been published (Supplementary Table 1). In this work, we report a 27-autopsy series with the longest illness duration (median 39 days, ranging 9–108 days) in which viral detection has been performed in post-mortem lung samples, including those with longer disease duration (95 and 108 days). For this reason, this work provides an opportunity to elucidate RNA SARS-CoV-2 persistence in patients with critical illness. According to Supplementary Table 1, only 17 patients in previously reported series showed viral RNA in lung samples after 4 weeks of illness (between 29 and 64 days). The high rate of positivity in our series could be related to the fact that we obtained samples from all lung lobes, a procedure performed in only a few of the previous studies (Supplementary Table 1).

We detected sgRNA in 14 of 26 (53.8%) adult patients, including two who died after 49 and 57 days of disease. These results are in accordance with the observations of Dorward et al. (2021), who detected sgRNA in a post-mortem lung sample from one patient who died 42 days after the onset of illness. Other studies evaluating sgRNA in autopsy samples were limited to patients with less than 27 days of disease (Desai et al., 2020; Bhatnagar et al., 2021). The role of sgRNA detection as a marker of viral replication is a matter of debate. Whereas different authors have reported sgRNA as a demonstration of persistent viral replication (Santos Bravo et al., 2021), other authors have questioned this claim (Alexandersen et al., 2020; Verma et al., 2021).

We did not detect SARS-Cov-2 mRNA in any organ but the lung in patients with more than 2 weeks of evolution, and no other organs presented lesions related with SARS-Cov-2 infection but the lungs. These facts support the role of persistent lung viral shedding in lung pathology. Moreover, it might suggest a rapid and short extrapulmonary spread of SARS-CoV-2 in the acute phase of the disease, which is generally cleared without triggering any explosive local inflammatory response in other organs (Ferrer-Gómez et al., 2021; Skok et al., 2021).

Persistent RNA viral shedding could be related to host immunity in severe COVID-19 cases, which is supported by the results observed in our pediatric patient with severe primary immunodeficiency. This patient is similar to a few previously reported living patients with secondary immunodeficiency in whom ongoing viral replication was demonstrated for more than 100 days (Avanzato et al., 2020; Baang et al., 2021; Truong et al., 2021). In our case, we were able to demonstrate active viral replication not only by detecting sgRNA with RT-PCR, but also by detecting viral protein and mRNA using IHC and ISH, respectively. As far as we know, this is the only patient reported so far in whom active SARS-CoV-2 viral replication has been demonstrated in the lung after 3 months from the onset of disease. This observation could have important implications for the antiviral treatment of immunocompromised COVID-19 patients with severe lung disease.

The immunocompromised populations, including immunosenescent elderly patients or patients subjected to immunosuppression treatment – which most of the patients in this series were (Table 1), were probably unable to completely eliminate viral particles and the vicious cycle of viral replication and inflammatory response persisted, contributing to chronic deterioration of the lung tissues (Avanzato et al., 2020; Reuken et al., 2021). These observations about viral persistence could have important implications in the selection of new intra-host viral variants (Truong et al., 2021) and consequently the effectiveness of vaccine or antiviral treatment of immunocompromised COVID-19 patients (Sulaiman et al., 2021; Tsukada et al., 2021). The different pandemic waves have been related to the description of new SARS-CoV-2 variants. This raises the question on whether our ISH and IHQ techniques, based on the detection of the spike RNA and protein, respectively, could recognize SARS-CoV-2 variants of concern. However, our study was carried out during the first three pandemic waves, when the SARS-CoV-2 variants B.1, B.1.177 and B.1.1.7 were successively dominant (Hodcroft et al., 2021; López et al., 2021; Martínez-García et al., 2021; Viedma et al., 2021).

Patients hospitalized with COVID-19 have a high risk of secondary infections due to intensive care unit (ICU) admission, prolonged mechanical ventilation, and severe lymphopenia (Ripa et al., 2021). In our series, 13 patients showed secondary pulmonary infections. The frequency of acute bronchopneumonia in our series (42.31%) was similar to that found in the largest autopsy series published so far, in which the authors found superimposed acute bronchopneumonia in 45 out of 82 patients (55%).

It has been suggested that COVID-19–associated pulmonary aspergillosis (CAPA) is a contributing factor to mortality in patients with severe COVID-19 (Salazar et al., 2021). In our series, 7.69% of the patients showed CAPA. The true incidence of CAPA has not been well established since its diagnosis is challenging from a clinical point of view (Koehler et al., 2021). The frequency of proven CAPA in autopsies varies among series. Thus, among the 24 reviewed series in Supplementary Table 1, only 3 series reported cases of CAPA, with a frequency ranging from 3.8 to 38.1% (Schurink et al., 2020; Berezowska et al., 2021; Yao et al., 2021). Similarly, whereas (Bryce et al., 2021) reported only 2 patients with CAPA in their series of 100 patients (2%), (Fortarezza et al., 2021) reported 9 CAPA cases among 45 autopsies (20%). These authors observed that CAPA increased mainly in the second wave of the pandemic (7 out of 17 vs. 2 out of 28 during the first wave), which was attributed to a higher use of corticosteroids during the second wave.

Corticosteroid use is a known risk factor for CMV reactivation and disease. In our series, 2 patients (7.69%) showed CMV lung infection. Niitsu et al investigated the frequency and characteristics of CMV infection in 26 critically ill patients with COVID-19 who required mechanical ventilation for more than 1 week (Niitsu et al., 2021). They found that one in four patients developed CMV infection during mechanical ventilation and one patient died from CMV pneumonia. CMV infection was associated with lymphopenia on ICU admission, prolonged mechanical ventilation, and increased mortality. Similar results have been reported in other clinical series (Yamamoto et al., 2021). CMV infection was not reported in any of the 24 autopsy series we reviewed.

In the pediatric patient in this series, we demonstrated the presence of Polyomavirus BK inclusions in epithelial cells using IHC and ultrastructural studies. Pulmonary lesions produced by Polyomavirus BK seem to be very infrequent (Cubukcu-Dimopulo et al., 2000). As far as we know, this is the first case of pulmonary infection by Polyomavirus BK reported in a COVID-19 patient. However, COVID-19 infection has been considered a risk factor for an increasing number of Polyomavirus BK infections in recipients of kidney transplantation (Meshram et al., 2021).

Our study has several limitations. The number of patients is not very high. Moreover, preanalytical issues related with FFPE samples could have negatively affected RNA detection. Finally, we could not test virus infectivity in any sample due to the lack of appropriate biosecure facilities. Despite these limitations, our results suggest a role of viral prolonged presence in the persistence of severe lung lesions in COVID-19 patients and of host immunity in the clearance of viral infection, given that immunocompromised patients are more susceptible to prolonged viral infections. Moreover, pulmonary co-infections may have an impact in the prognosis of these patients.

## Data Availability Statement

The raw data supporting the conclusions of this article will be made available by the authors, without undue reservation.

## Ethics Statement

The studies involving human participants were reviewed and approved by the Research Ethics Committee, Hospital Universitario Ramón y Cajal. Written informed consent to participate in this study was provided by the participants’ legal guardian/next of kin. Written informed consent was obtained from the minor(s)’ legal guardian/next of kin for the publication of any potentially identifiable images or data included in this article.

## Author Contributions

JP, JG, and BP-M contributed to the conception of the study. TC-C, LM-G, ASn, and MR contributed to the sample processing. MA-R, NM-D-C, IR-C, RP, ASi, RR, and JR-P contributed to the data collection. DP, JP, JG, BP-M, TC-C, and LM-G contributed to the data analysis and interpretation. JP, JG, and IC-B drafted the manuscript. All authors revised the manuscript for intellectual content and approved the final version of the manuscript.

## Conflict of Interest

The authors declare that the research was conducted in the absence of any commercial or financial relationships that could be construed as a potential conflict of interest.

## Publisher’s Note

All claims expressed in this article are solely those of the authors and do not necessarily represent those of their affiliated organizations, or those of the publisher, the editors and the reviewers. Any product that may be evaluated in this article, or claim that may be made by its manufacturer, is not guaranteed or endorsed by the publisher.

## References

[B1] Alexandersen S. Chamings A. Bhatta T. R. (2020). SARS-CoV-2 genomic and subgenomic RNAs in diagnostic samples are not an indicator of active replication. *Nat. Commun.* 11:6059. 10.1038/s41467-020-19883-7 33247099 PMC7695715

[B2] Avanzato V. A. Matson M. J. Seifert S. N. Pryce R. Williamson B. N. Anzick S. L. (2020). Case study: prolonged infectious SARS-CoV-2 shedding from an asymptomatic immunocompromised individual with cancer. *Cell* 183 1901–1912.e9. 10.1016/j.cell.2020.10.049 33248470 PMC7640888

[B3] Baang J. H. Smith C. Mirabelli C. Valesano A. L. Manthei D. M. Bachman M. A. (2021). Prolonged severe acute respiratory syndrome coronavirus 2 replication in an immunocompromised patient. *J. Infect. Dis.* 223 23–27. 10.1093/infdis/jiaa666 33089317 PMC7797758

[B4] Berezowska S. Lefort K. Ioannidou K. Ndiaye D.-R. Maison D. Petrovas C. (2021). Postmortem cardiopulmonary pathology in patients with COVID-19 infection: single-center report of 12 autopsies from Lausanne, Switzerland. *Diagnostics* 11:1357. 10.3390/diagnostics11081357 34441292 PMC8393761

[B5] Bhatnagar J. Gary J. Reagan-Steiner S. Estetter L. B. Tong S. Tao Y. (2021). Evidence of severe acute respiratory syndrome coronavirus 2 replication and tropism in the lungs, airways, and vascular endothelium of patients with fatal coronavirus disease 2019: an autopsy case series. *J. Infect. Dis.* 223 752–764. 10.1093/infdis/jiab039 33502471 PMC7928839

[B6] Bryce C. Grimes Z. Pujadas E. Ahuja S. Beasley M. B. Albrecht R. (2021). Pathophysiology of SARS-CoV-2: the mount sinai COVID-19 autopsy experience. *Mod. Pathol.* 34 1456–1467. 10.1038/s41379-021-00793-y 33795830 PMC8015313

[B7] Cubukcu-Dimopulo O. Greco A. Kumar A. Karluk D. Mittal K. Jagirdar J. (2000). BK virus infection in AIDS. *Am. J. Surg. Pathol.* 24 145–149. 10.1097/00000478-200001000-00019 10632500

[B8] Desai N. Neyaz A. Szabolcs A. Shih A. R. Chen J. H. Thapar V. (2020). Temporal and spatial heterogeneity of host response to SARS-CoV-2 pulmonary infection. *Nat. Commun.* 11:6319. 10.1038/s41467-020-20139-7 33298930 PMC7725958

[B9] Dorward D. A. Russell C. D. Um I. H. Elshani M. Armstrong S. D. Penrice-Randal R. (2021). Tissue-Specific Immunopathology in Fatal COVID-19. *Am. J. Respir. Crit. Care Med.* 203 192–201. 10.1164/rccm.202008-3265OC 33217246 PMC7874430

[B10] Evert K. Dienemann T. Brochhausen C. Lunz D. Lubnow M. Ritzka M. (2021). Autopsy findings after long-term treatment of COVID-19 patients with microbiological correlation. *Virchows Arch*. 479 97–108. 10.1007/s00428-020-03014-0 33471172 PMC7816067

[B11] Ferrer-Gómez A. Pian-Arias H. Carretero-Barrio I. Navarro-Cantero A. Pestaña D. de Pablo R. (2021). Late cardiac pathology in severe covid-19. A postmortem series of 30 patients. *Front. Cardiovasc. Med.* 8:1372. 10.3389/fcvm.2021.748396 34722679 PMC8555828

[B12] Fortarezza F. Boscolo A. Pezzuto F. Lunardi F. Jesús Acosta M. Giraudo C. (2021). Proven COVID-19-associated pulmonary aspergillosis in patients with severe respiratory failure. *Mycoses* 64 1223–1229. 10.1111/myc.13342 34157166 PMC8446949

[B13] Hodcroft E. B. Zuber M. Nadeau S. Vaughan T. G. Crawford K. H. D. Althaus C. L. (2021). Spread of a SARS-CoV-2 variant through Europe in the summer of 2020. *Nature* 595 707–712. 10.1038/s41586-021-03677-y 34098568

[B14] Koehler P. Bassetti M. Chakrabarti A. Chen S. C. A. Colombo A. L. Hoenigl M. (2021). Defining and managing COVID-19-associated pulmonary aspergillosis: the 2020 ECMM/ISHAM consensus criteria for research and clinical guidance. *Lancet Infect. Dis.* 21 e149–e162. 10.1016/S1473-3099(20)30847-1 33333012 PMC7833078

[B15] Li Y. Wu J. Wang S. Li X. Zhou J. Huang B. (2021). Progression to fibrosing diffuse alveolar damage in a series of 30 minimally invasive autopsies with COVID-19 pneumonia in Wuhan, China. *Histopathology* 78 542–555. 10.1111/his.14249 32926596 PMC8848295

[B16] López M. G. Chiner-Oms Á García de Viedma D. Ruiz-Rodriguez P. Bracho M. A. Cancino-Muñoz I. (2021). The first wave of the COVID-19 epidemic in Spain was associated with early introductions and fast spread of a dominating genetic variant. *Nat. Genet.* 53 1405–1414. 10.1038/s41588-021-00936-6 34594042 PMC8481935

[B17] Martínez-García L. Espinel M. A. Abreu M. González-Alba J. M. Gijón D. McGee A. (2021). Emergence and spread of B.1.1.7 lineage in primary care and clinical impact in the morbi-mortality among hospitalized patients in Madrid, Spain. *Microorganisms* 9:1517. 10.3390/microorganisms9071517 34361951 PMC8307589

[B18] Meshram H. S. Kute V. B. Chauhan S. (2021). BK polyomavirus infection following COVID-19 infection in renal transplant recipients: a single-center experience. *Kidney Res. Clin. Pract.* 40 496–500. 10.23876/j.krcp.21.082 34370932 PMC8476307

[B19] Niitsu T. Shiroyama T. Hirata H. Noda Y. Adachi Y. Enomoto T. (2021). Cytomegalovirus infection in critically ill patients with COVID-19. *J. Infect.* 83 496–522. 10.1016/j.jinf.2021.07.004 34252496 PMC8268671

[B20] Pérez-Mies B. Gómez-Rojo M. Carretero-Barrio I. Bardi T. Benito A. García-Cosío M. (2021). Pulmonary vascular proliferation in patients with severe COVID-19: an autopsy study. *Thorax* 76 1044–1046. 10.1136/thoraxjnl-2020-216714 33758071 PMC7992389

[B21] Reuken P. A. Stallmach A. Pletz M. W. Brandt C. Andreas N. Hahnfeld S. (2021). Severe clinical relapse in an immunocompromised host with persistent SARS-CoV-2 infection. *Leukemia* 35 920–923. 10.1038/s41375-021-01175-8 33608636 PMC7893131

[B22] Ripa M. Galli L. Poli A. Oltolini C. Spagnuolo V. Mastrangelo A. (2021). Secondary infections in patients hospitalized with COVID-19: incidence and predictive factors. *Clin. Microbiol. Infect.* 27 451–457. 10.1016/j.cmi.2020.10.021 33223114 PMC7584496

[B23] Salazar F. Bignell E. Brown G. D. Cook P. C. Warris A. (2021). Pathogenesis of respiratory viral and fungal coinfections. *Clin. Microbiol. Rev.* 35:e00094-21. 10.1128/CMR.00094-21 34788127 PMC8597983

[B24] Santos Bravo M. Nicolás D. Berengua C. Fernandez M. Hurtado J. C. Tortajada M. (2021). Severe acute respiratory syndrome coronavirus 2 normalized viral loads and subgenomic RNA detection as tools for improving clinical decision making and work reincorporation. *J. Infect. Dis.* 224 1325–1332. 10.1093/infdis/jiab394 34329473 PMC8436374

[B25] Schaefer I.-M. Padera R. F. Solomon I. H. Kanjilal S. Hammer M. M. Hornick J. L. (2020). In situ detection of SARS-CoV-2 in lungs and airways of patients with COVID-19. *Mod. Pathol.* 33 2104–2114. 10.1038/s41379-020-0595-z 32561849 PMC7304376

[B26] Schurink B. Roos E. Radonic T. Barbe E. Bouman C. S. C. de Boer H. H. (2020). Viral presence and immunopathology in patients with lethal COVID-19: a prospective autopsy cohort study. *Lancet Microbe* 1 e290–e299. 10.1016/S2666-5247(20)30144-0 33015653 PMC7518879

[B27] Siddiqi H. K. Mehra M. R. (2020). COVID-19 illness in native and immunosuppressed states: a clinical-therapeutic staging proposal. *J. Heart Lung Transplant.* 39 405–407. 10.1016/j.healun.2020.03.012 32362390 PMC7118652

[B28] Skok K. Stelzl E. Trauner M. Kessler H. H. Lax S. F. (2021). Post-mortem viral dynamics and tropism in COVID-19 patients in correlation with organ damage. *Virchows Arch.* 478 343–353. 10.1007/s00428-020-02903-8 32815036 PMC7438212

[B29] Sulaiman I. Chung M. Angel L. Tsay J.-C. J. Wu B. G. Yeung S. T. (2021). Microbial signatures in the lower airways of mechanically ventilated COVID-19 patients associated with poor clinical outcome. *Nat. Microbiol.* 6 1245–1258. 10.1038/s41564-021-00961-5 34465900 PMC8484067

[B30] The COVID-19 Autopsy Project (2020). The first COVID-19 autopsy in Spain performed during the early stages of the pandemic. *Rev. Española Patol.* 53 182–187. 10.1016/j.patol.2020.05.004 32650969 PMC7245282

[B31] Truong T. T. Ryutov A. Pandey U. Yee R. Goldberg L. Bhojwani D. (2021). Increased viral variants in children and young adults with impaired humoral immunity and persistent SARS-CoV-2 infection: a consecutive case series. *EBioMedicine* 67:103355. 10.1016/j.ebiom.2021.103355 33915337 PMC8072072

[B32] Tsukada A. Suzuki M. Kishino Y. Misumi K. Igari T. Nakajima N. (2021). A kidney transplant patient who died of COVID-19-associated severe acute respiratory distress syndrome. *Intern. Med.* 60 2297–2300. 10.2169/internalmedicine.7089-21 34053986 PMC8355385

[B33] Verma R. Kim E. Martínez-Colón G. J. Jagannathan P. Rustagi A. Parsonnet J. (2021). SARS-CoV-2 subgenomic RNA kinetics in longitudinal clinical samples. *Open Forum Infect. Dis.* 8:ofab310. 10.1093/ofid/ofab310 34295944 PMC8291522

[B34] Viedma E. Dahdouh E. González-Alba J. González-Bodi S. Martínez-García L. Lázaro-Perona F. (2021). Genomic epidemiology of SARS-CoV-2 in Madrid, Spain, during the first wave of the pandemic: fast spread and early dominance by D614G variants. *Microorganisms* 9:454. 10.3390/microorganisms9020454 33671631 PMC7926973

[B35] Yamamoto Y. Shiroyama T. Hirata H. Kuge T. Matsumoto K. Yoneda M. (2021). Prolonged corticosteroid therapy and cytomegalovirus infection in patients with severe COVID-19. *J. Med. Virol.* 1–7. 10.1002/jmv.27421 [Epub ahead of print]. 34708883 PMC8661974

[B36] Yao X.-H. Luo T. Shi Y. He Z.-C. Tang R. Zhang P.-P. (2021). A cohort autopsy study defines COVID-19 systemic pathogenesis. *Cell Res.* 31 836–846. 10.1038/s41422-021-00523-8 34135479 PMC8208380

