# Supplementary material for: RNA SARS-CoV-2 Persistence in the Lung of Severe COVID-19 Patients: A Case Series of Autopsies
Source: Front Microbiol. 2022 Jan 31;13:824967. doi: 10.3389/fmicb.2022.824967 (PMC8841799; doi:10.3389/fmicb.2022.824967)
Supplement: Supplementary file 1 [file Table_1.DOCX]

**Supplemental table 1:** Autopsy series with at least 4 patients in which Sars-Cov2 has been detected in post-mortem lung samples using different methodologies.

| **Series** | **Duration of illness (median)** | **Range** | **Number of patients studied** | **Number of patients with positive lung samples** | **Number of patients with positive lung samples after 4 weeks** | **Duration of illness of the patient with the longest evolution** |
| --- | --- | --- | --- | --- | --- | --- |
| Present series* | 39 | 9-108 | 26 | 25 | 20 | 108 |
| (Menter et al., 2020) | 5.7 | 0-16 | 21 | 20 | - | 16 |
| (Bussani et al., 2020) | 6 | 1-29 | 11 | 10 | 1 | 29 |
| (Bradley et al., 2020) | 7 | 1-14 | 12 | 12 | - | 14 |
| (Bhatnagar et al., 2021) | 7 | 1-26 | 64 | 32 | - | 26 |
| (Schaller et al., 2020) | 7.5 | 1-26 | 10 | 10 | - | 26 |
| (El Jamal et al., 2021)* | 7.5 | 1-34 | 14 | 4 | 1 | 34 |
| (Lax et al., 2020) ^#^ | 9 | 4-18 | 11 | 11 | - | 18 |
| **Series** | **Duration of illness (median)** | **Range** | **Number of patients studied** | **Number of patients with positive lung samples** | **Number of patients with positive lung samples after 4 weeks** | **Duration of illness of the patient with the longest evolution** |
| (Hirschbühl et al., 2021)* | 11 | 1-36 | 19 | 19 | - | 36 |
| (Deinhardt-Emmer et al., 2021)* | 11.64 | 4-30 | 11 | 10 | 1 | 30 |
| (Schurink et al., 2020) | 12 | 5-44 | 21 | 8 | 1 | 31 |
| (Skok et al., 2021)* | 12.3 | 4-36 | 19 | 17 | 1 | 34 |
|  |  |  |  |  |  |  |
| (Bösmüller et al., 2020) | 12.5 | 1-35 | 4 | 4 | 1 | 35 |
| (Recalde-Zamacona et al., 2020) | 13 | 8-25 | 10 | 8 | - | - |
| (Remmelink et al., 2020)* | 13 | 3-31 | 17 | 6 | 1 | 31 |
| (Hanley et al., 2020) | 14 | 9-27 | 5 | 5 | - | 27 |
|  |  |  |  |  |  |  |
| **Series** | **Duration of illness (median)** | **Range** | **Number of patients studied** | **Number of patients with positive lung samples** | **Number of patients with positive lung samples after 4 weeks** | **Duration of illness of the patient with the longest evolution** |
| (Sauter et al., 2020) | 14.5 | 1-25 | 8 | 3 | - | 9 |
| (Berezowska et al., 2021) | 14.5 | 6-38 | 12 | 12 | - | 38 |
| (Roden et al., 2021)* | 16 | 3-100 | 12 | 11 | 2 | 55 |
| (Dell’Aquila et al., 2020) | 18 | 8-39 | 12 | 5 | - | 29 |
| (Wylezich et al., 2021)* | 18.38 | 11-30 | 8* | 6 | 0 | 19 |
| (Barisione et al., 2021) | 21.5 | 4-35 | 8 | 2 | - | 9 |
| (Dorward et al., 2021)* | 23 | 9-42 | 11 | 11 | 2 | 42 |
| (Wong et al., 2021)* | 31 | 12-64 | 8 | 8 | 4 | 64 |
|  |  |  |  |  |  |  |
| **Series** | **Duration of illness (median)** | **Range** | **Number of patients studied** | **Number of patients with positive lung samples** | **Number of patients with positive lung samples after 4 weeks** | **Duration of illness of the patient with the longest evolution** |
| (Yao et al., 2021)* | 38.5 | 30-52 | 26 | 24 | - | - |

*Multiple lobes per lung studied.

^#^Eight patients of this study were included in (Skok et al., 2021).
